# Our Hospital Articles

**Published:** 1860-02

**Authors:** 


*Our Hospital Articles.*—We are unable this month to furnish our regular hospital article; but they will be continued till all the available institutions have been described, when we propose to make an abstract of all of them.

